# Promoter Methylation of *PRKCB*, *ADAMTS12*, and *NAALAD2* Is Specific to Prostate Cancer and Predicts Biochemical Disease Recurrence

**DOI:** 10.3390/ijms22116091

**Published:** 2021-06-05

**Authors:** Kristina Daniunaite, Arnas Bakavicius, Kristina Zukauskaite, Ieva Rauluseviciute, Juozas Rimantas Lazutka, Albertas Ulys, Feliksas Jankevicius, Sonata Jarmalaite

**Affiliations:** 1 Life Sciences Center, Institute of Biosciences, Vilnius University, 10257 Vilnius, Lithuania; vailaomalyn@yahoo.com (K.D.); ieva.rauluseviciute@gmail.com (I.R.); juozas.lazutka@gf.vu.lt (J.R.L.); 2 National Cancer Institute, 08660 Vilnius, Lithuania; arnas.bakavicius@gmail.com (A.B.); kristinazu@gmail.com (K.Z.); albertasulys@gmail.com (A.U.); Feliksas.Jankevicius@santa.lt (F.J.); 3 Centre of Urology, Vilnius University Hospital Santaros Klinikos, 08661 Vilnius, Lithuania; 4 Faculty of Medicine, Vilnius University, 03101 Vilnius, Lithuania

**Keywords:** prostate cancer, biochemical recurrence, DNA methylation, *ADAMTS12*, *NAALAD2*, *PRKCB*

## Abstract

The molecular diversity of prostate cancer (PCa) has been demonstrated by recent genome-wide studies, proposing a significant number of different molecular markers. However, only a few of them have been transferred into clinical practice so far. The present study aimed to identify and validate novel DNA methylation biomarkers for PCa diagnosis and prognosis. Microarray-based methylome data of well-characterized cancerous and noncancerous prostate tissue (NPT) pairs was used for the initial screening. Ten protein-coding genes were selected for validation in a set of 151 PCa, 51 NPT, as well as 17 benign prostatic hyperplasia samples. The Prostate Cancer Dataset (PRAD) of The Cancer Genome Atlas (TCGA) was utilized for independent validation of our findings. Methylation frequencies of *ADAMTS12*, *CCDC181*, *FILIP1L*, *NAALAD2*, *PRKCB,* and *ZMIZ1* were up to 91% in our study. PCa specific methylation of *ADAMTS12*, *CCDC181*, *NAALAD2,* and *PRKCB* was demonstrated by qualitative and quantitative means (all *p* < 0.05). In agreement with PRAD, promoter methylation of these four genes was associated with the transcript down-regulation in the Lithuanian cohort (all *p* < 0.05). Methylation of *ADAMTS12*, *NAALAD2,* and *PRKCB* was independently predictive for biochemical disease recurrence, while *NAALAD2* and *PRKCB* increased the prognostic power of multivariate models (all *p* < 0.01). The present study identified methylation of *ADAMTS12*, *NAALAD2,* and *PRKCB* as novel diagnostic and prognostic PCa biomarkers that might guide treatment decisions in clinical practice.

## 1. Introduction

Despite tremendous improvements in diagnostics and treatment tactics, prostate cancer (PCa) is the second most common malignancy among men and the leading cause of cancer-related death among all urogenital cancers [1]. Timely diagnosis of the disease is essential for selecting the most appropriate treatment strategy and successful disease management. After introducing aggressive prostate-specific antigen (PSA) testing into clinical practice, the diagnosis of PCa has dramatically shifted towards the early stage and localized disease [2]. However, limited PSA prognostic power has increased the detection of clinically insignificant disease, leading to over-treatment with a huge negative impact on males’ physical and mental well-being [3]. All these drawbacks encouraged the search for new diagnostic and prognostic PCa biomarkers with better performance characteristics.

Aberrant promoter methylation is an early event in carcinogenesis and has been extensively studied in the development of PCa [4]. Methylation of promoter region has been reported to be responsible for silencing more than 100 PCa genes, where glutathione S-transferase pi 1 (*GSTP1*) and RAS association domain family member 1 (*RASSF1*) has been the most intensively analyzed [4]. Recent genome-wide studies have revealed novel insight into the epigenetic landscape of PCa [5,6,7,8,9] and suggested new diagnostic and prognostic biomarkers. However, only a small part of them have been transferred into clinical practice so far [5,7,8]. Scientific evidence about prognostic DNA methylation biomarkers remains scarce as most investigations have resulted in ambiguous data. Besides, most of these studies have focused on comparing cancerous with noncancerous prostate tissue (NPT) rather than focusing on disease aggressiveness. However, aberrant DNA methylation is a promising source for PCa biomarkers, so thorough screening and consecutive validation in independent cohorts are needed to identify the most prospective ones for clinical usage.

The present study aimed at the identification and validation of novel DNA methylation biomarkers for PCa diagnosis and prognosis. Initially, 10 protein-coding genes were selected from previously reported microarray-based methylome screening [10] for promoter methylation analysis in PCa and control samples. For validation, a 2-step approach was used utilizing the Prostate Cancer Dataset (PRAD) of The Cancer Genome Atlas (TCGA) as an independent validation cohort [9]. Methylated promoter status was further associated with downregulated gene expression and clinico-pathological characteristics of PCa patients. Survival analysis was performed to evaluate the prognostic potential of the epigenetic biomarkers.

## 2. Materials and Methods

### 2.1. Patients and Samples

Treatment-naïve patients (*N* = 151) with histologically confirmed PCa who underwent radical prostatectomy (RP) at Vilnius University Hospital Santaros Klinikos between January 2008 and December 2014 were included. Cancerous (*N* = 151) and noncancerous prostate tissue (*N* = 51) samples were collected from the definitive pathology of these patients. As a control group, 17 benign prostatic hyperplasia (BPH) samples, obtained from open prostatectomy material due to BPH, were included in the study. All tissues were dissected and evaluated by an expert pathologist, as reported previously [11]. Approval from the Lithuanian Bioethics Committee was obtained, and written informed consent was obtained from all participants.

Biochemical disease recurrence (BCR) following RP was defined as a postoperative PSA > 0.2 ng/mL with a subsequent confirmatory value [12]. Full follow-up data were available for 88.7% (134/151) of patients with a mean follow-up of 3.3 years. Clinico-pathological and molecular characteristics of the study subsets are provided in Table S1.

### 2.2. DNA Methylation Microarray Data

For the screening step, genome-wide DNA methylation profiling data of 9 paired PCa and NPT samples (GEO identifier GSE89243), reported in our previous study [10], has been reanalyzed to identify potential PCa biomarkers. Samples were processed according to the manufacturer’s protocol using two-color Human DNA Methylation 1 × 244 K Microarrays, which interrogate 27,627 known CpG islands (Agilent Technologies, Santa Clara, CA, USA). Saturated, non-uniform, and outlier probe signals were excluded from the analysis. The Cy3/Cy5 fluorescence ratios representing methylated/reference DNA were calculated and normalized to obtain log_2_ values for further analysis. Probe annotations were uploaded from the SureDesign platform (https://earray.chem.agilent.com/suredesign; accessed 1 March 2018). They were updated using the UCSC Genome Browser (https://genome.ucsc.edu; accessed 1 March 2018) according to the latest version of the human reference genome (GRCh38). Probes that were not detected in ≥30% of all samples were filtered out. After additional group comparison-specific filtering, only probes detected in 100% of samples in at least 1 of 2 groups were compared. Fold change (FC) values were estimated, and a paired (where applicable) or unpaired t-test was applied. Calculations were performed with GeneSpring GX v14.5 software (Agilent Technologies).

The gene set enrichment analysis (GSEA) was performed using the publicly accessible online GSEA tool and Molecular Signatures Database (MSigDB, v5.2; http://software.broadinstitute.org/gsea; accessed 1 March 2018) [13]. The collection of 50 hallmark gene sets, which conveys a specific biological state or process and displays the coherent expression, were utilized for GSEA [14]. False discovery rate (FDR) q-value with the cut-off <0.05 was used to correct for multiple testing.

### 2.3. DNA Purification and Qualitative Methylation Analysis

DNA was extracted from fresh-frozen tissue samples following the standard phenol-chloroform purification protocol as described previously [10]. Four hundred ng of purified DNA were bisulfite-modified, using the EZ DNA Methylation™ Kit (Zymo Research, Irvine, CA, USA) following the manufacturer’s instructions, and analyzed by methylation-specific PCR (MSP). MSP primers, overlapping with the location of the microarray probes of interest, were designed with Methyl Primer Express Software v1.0 (Applied Biosystems™, Thermo Fisher Scientific, Carlsbad, CA, USA) and ordered from Metabion (Martinsried, Germany) (Table S2). The reaction mix (25 μL) consisted of 1× Maxima Hot Start Taq PCR buffer, 2.5 mM MgCl_2_, 0.4 mM of each dNTP, 1.25 U Maxima Hot Start Taq DNA Polymerase (all from Thermo Scientific™, Thermo Fisher Scientific, Vilnius, Lithuania), 1 µM of each primer, and 10–20 ng of bisulfite-treated DNA. Reaction conditions were optimized before the study and included 35–38 cycles with primer annealing step 55–62 °C for 45 s (Table S2). Methylation-positive (MC), methylation-negative, and non-template controls (NTC) were included in each MSP assay.

### 2.4. Quantitative Methylation Analysis

Target-specific quantitative MSP (QMSP) primers and hydrolysis probes for *PRKCB*, *CCDC181*, *ADAMTS12*, and *NAALAD2* were designed using MethPrimer software v1.0 (http://www.urogene.org/methprimer; accessed 1 March 2018) [15]. Stably expressed *ACTB* served as a normalization gene, which was included in each assay to remove the non-biological variation. Primer sequences are provided in Table S3. QMSP was performed with technical triplicates for each set of primers. The reaction mixture of a 20-μL final volume contained 1× TaqMan^®^ Universal Master Mix II, no UNG (Applied Biosystems™), 300 nM of each primer, 50 nM of TaqMan probe, and ~10 ng of bisulfite-converted DNA. QMSP was performed using the Mx3005P qPCR System (Agilent Technologies) under the following regime: 95 °C for 10 min followed by 50 cycles of 95 °C for 15 s and 60 °C for 1 min. When routinely included MCs gave a positive signal, a run was considered valid, and there was no amplification in NTC wells. The methylation level of an individual gene was estimated based on ΔΔCq algorithm and expressed as a percentage of the MC. Samples with a methylation level of >0.1% were considered methylated when analyzed qualitatively.

### 2.5. RNA Extraction and Gene Expression Analysis by RT-qPCR

Total RNA samples, prepared during our previous study [10] from the same patient cohort, were used for target gene expression analysis by quantitative PCR (qPCR). Briefly, the mirVana™ miRNA Isolation Kit (Ambion, Thermo Fisher Scientific, Foster City, CA, USA) was used for total RNA extraction. Only samples having high purity parameters and RNA integrity number (RIN) ≥ 7, as measured using the 2100 Bioanalyzer (Agilent Technologies), were submitted for further analysis (Table S1). Reverse transcription (RT) was carried out using a High Capacity cDNA Reverse Transcription Kit with RNase Inhibitor (Applied Biosystems™), and 250 ng of RNA as a template.

Expression of genes *PRKCB*, *CCDC181*, *ADAMTS12*, *NAALAD2*, *ZMIZ1*, and endogenous control *HPRT1* was evaluated using primers and probes from Applied Biosystems (TaqMan^®^ Gene Expression Assays). RT-qPCR was performed with technical triplicates for each set of primers/probes. The reaction mixture of a 20-μL final volume contained 1× TaqMan^®^ Universal Master Mix II, no UNG (Applied Biosystems™), 0.6× of TaqMan^®^ assay, and 2 μL of cDNA, under the following conditions: 95 °C for 10 min, followed by 40 cycles of 95 °C for 15 s and 60 °C for 1 min. Real-time amplification was achieved using the Mx3005P qPCR System (Agilent Technologies). Multiple NTCs were included in each RT-qPCR run. Data pre-processing was performed with GenEx v6.0.1 software (MultiD Analyses AB, Göteburg, Sweden): target gene expression levels were normalized with *HPRT1.* The obtained cycle of quantification differences (ΔCq values) was converted to an arbitrary linear scale (2^−ΔCq^). The obtained relative gene expression values were used for further analysis.

### 2.6. Statistical Analysis

Statistical analyses were performed using STATISTICA™ v8.0 (StatSoft, Tulsa, OK, USA) and MedCalc^®^ v12.7 (MedCalc Software, Ostend, Belgium). The methylation frequency was calculated as the percentage of samples in which methylation of a particular gene was detected. Student’s *t*-test or Mann-Whitney U test was used to compare quantitative variables, while a 2-sided Fisher’s exact test was applied to compare categorical variables. Pearson (RP) and/or Spearman‘s (RS) rank correlation coefficients were calculated to test the associations between two variables. For BCR-free analysis, Kaplan-Meier curves with the log-rank test and Cox proportional hazards models were used. Parametric tests were applied for the analysis of TCGA data. Results were considered statistically significant when the *p*-value was <0.0500.

## 3. Results

### 3.1. Microarray-Based DNA Methylation Analysis

**Figure 1:**
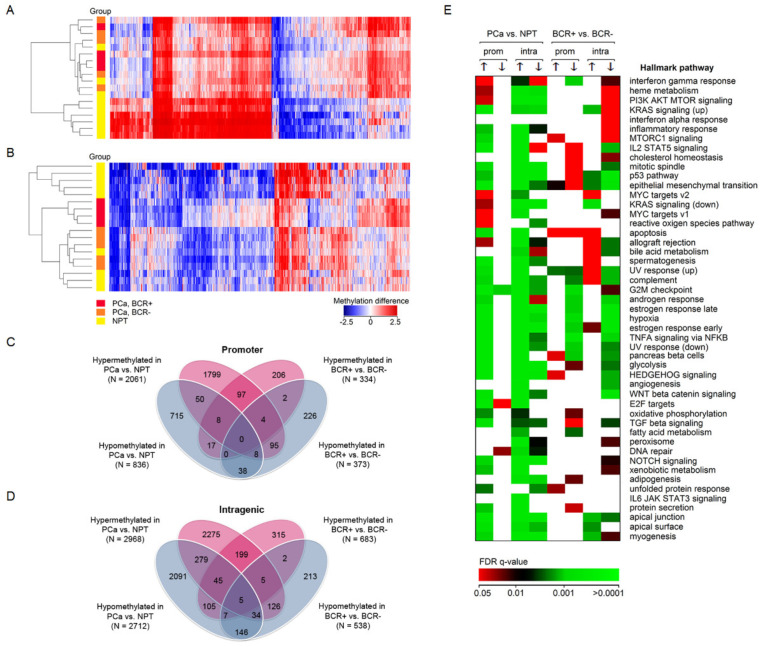
Microarray-based DNA methylation analysis. (**A**) DNA methylation differences between cancerous and NPT; (**B**) DNA methylation differences according to biochemical disease recurrence; (**C**,**D**) Venn diagrams of methylated genes in promoter and intragenic regions, according to tissue histology and BCR status; (**E**) Gene set enrichment analysis (GSEA) of differentially methylated genes. The color scale is the same for both heatmaps. The collection of Hallmark gene sets as defined in MSigDB were selected for GSEA. The empty cells in the graph represent insignificant q-values. PCa—prostate cancer; NPT—noncancerous prostate tissue; BCR+/−—biochemical disease recurrence, i.e., yes (+) or no (−); prom—promoter regions, intra—intragenic region; up/down arrows—gain/loss of methylation.

The global DNA methylation profile was analyzed in nine cancerous and NPT samples (Figure 1A–D) to evaluate the extent of epigenetic alterations in PCa. Significant methylation differences with FC ≥ 1.2 (*p* < 0.0500) were detected in 6899 genes, where 4227 (61.3%) and 3268 (47.4%) genes were hypermethylated and hypomethylated, respectively, including 596 (8.6%) overlapped genes (Figure 1A). The number of hypermethylated genes in promoter regions was much higher than hypomethylated changes (72.8% vs. 29.5%, respectively, with 2.3% overlap). Meanwhile, both alterations were similarly common in intragenic loci (55.8% vs. 51.0%, respectively, with 6.8% overlap; Figure 1C,D).

Less numerous DNA methylation differences were observed for patients with and without BCR (Figure 1B). Of 1804 genes with significant methylation differences, 969 (53.7%) and 868 (48.1%) genes were hypermethylated and hypomethylated, respectively, including 33 (1.8%) overlapped genes. Increase and decrease of methylation levels were similar in both promoter (53.2% vs. 47.6%, respectively, with 0.9% overlap) and intragenic regions (44.8% vs. 56.8%, respectively; Figure 1C,D).

Hypermethylation of 411 overlapped genes was detected comparing PCa vs. NPT and BCR positive vs. negative cases, while 291 genes were hypomethylated in both comparisons. Interestingly, some of these genes demonstrated hypermethylation and hypomethylation of different loci (Figure 1C,D). The top 50 genes with the most significant differences are provided in Table S4.

According to GSEA analysis, genes participating in cell cycle regulation, estrogen response, and apical junction were among the most significantly methylated in PCa vs. NPT (Figure 1E). Increased methylation levels were the most significantly different among genes participating in response to ultraviolet exposure and epithelial-mesenchymal transition. Hypomethylation was the most commonly detected in genes responsible for mitotic spindle formation and estrogen response. Similar genes were hypermethylated comparing BCR-positive vs. negative PCa cases, while genes associated with androgen and estrogen response, and hypoxia, were the most commonly hypomethylated (Figure 1E).

### 3.2. Promoter Methylation Analysis

**Figure 2:**
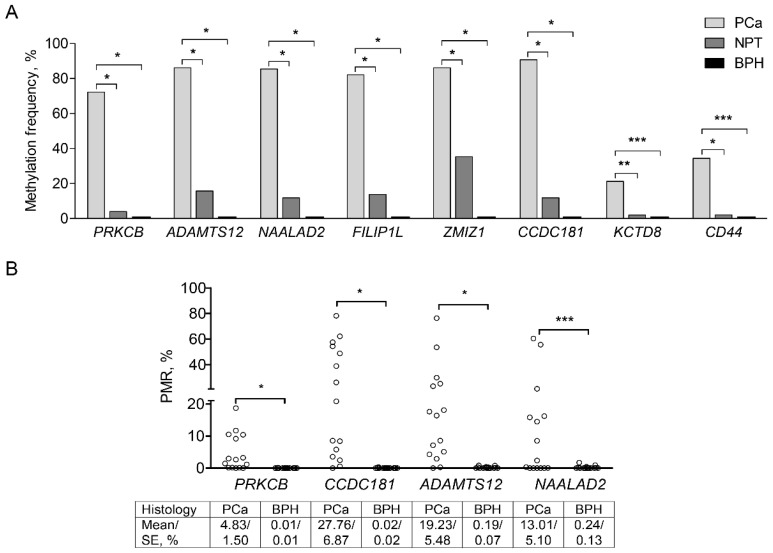
Promoter methylation analysis of the selected target genes in prostate tissue samples. (**A**) Promoter methylation frequencies of the analyzed genes (MSP data); (**B**) Methylation intensity values as a percentage of methylated reference (PMR) of genes *PRKCB*, *CCDC181*, *ADAMTS12*, and *NAALAD2* (QMSP data). Mean PMR values with standard error of the mean (SE) are given below for each group. PCa—prostate cancer, NPT—noncancerous prostate tissue, BPH—benign prostatic hyperplasia. Statistically significant *p*-values are marked in bold. *—*p* ≤ 0.0001, **—*p* < 0.001, ***—*p* < 0.05.

Ten genes (namely, *ADAMTS12*, *CCDC181*, *CD44*, *EPAS1*, *FILIP1L*, *KCTD8*, *NAALAD2*, *NEK9*, *PRKCB,* and *ZMIZ1*) were selected for further validation in 151 PCa, 51 NPT, and 17 BPH samples (Table S2), based on the methylation differences in promoter regions according to prostate tissue histology (*ADAMTS12*, *CCDC181*, *CD44*, *FILIP1L*, *KCTD8*, *NAALAD2*, *PRKCB*, *ZMIZ1*) or BCR status (*EPAS1*, *FILIP1L*, *NEK9*, *PRKCB*, *ZMIZ1*). Frequent methylation of *ADAMTS12*, *CCDC181*, *FILIP1L*, *NAALAD2*, *PRKCB,* and *ZMIZ1* (up to 90.7%), as well as less common of *CD44* and *KCTD8* (up to 34.4%), was identified in PCa, which significantly differed from NPT (0–35.3%) and BPH samples (0%) (all *p* < 0.0500; Figure 2A). Further investigation of *EPAS1* and *NEK9* was discontinued due to the lack of aberrant methylation events at the promoter regions in a subset of PCa and NPT samples. Quantitative analysis showed that methylation levels of *ADAMTS12*, *CCDC181*, *NAALAD2,* and *PRKCB* were significantly higher in randomly selected 15 PCa samples than 15 BPH samples (all *p* < 0.0500; Figure 2B).

To confirm our findings, 333 PCa cases from PRAD data collection of TCGA were used [9]. In line with our findings, all eight genes demonstrated significantly higher methylation levels in PCa than NPT (all *p* < 0.0001; Figure S1). Additionally, *CD44* (median β-value 0.26) and *KCTD8* (median β-value 0.15) were characterized by lower methylation levels in cancerous prostate tissue as compared to other genes (median β-values ≥ 0.48). In contrast, methylation levels of *FILIP1L* were relatively high in cancerous and NPT with median β-values of 0.89 and 0.84, respectively (Figure S1).

All selected genes were further analyzed according to clinico-pathological characteristics of the disease. Methylation frequency of *NAALAD2*, *PRKCB* and *ZMIZ1* significantly increased as the ISUP grade group increased, while *KCTD8* and *ZMIZ1* were more frequently methylated in locally advanced disease (≥pT3) (all *p* < 0.0500). Besides, *ADAMTS12*, *CD44, KCTD8,* and *PRKCB* were more commonly methylated in tumors harboring gene fusion between *TMPRSS2* and *ERG* (all *p* < 0.0500; Table S5). No associations between promoter methylation and PSA level, prostate volume, or the patient’s age were detected.

### 3.3. Gene Expression Analysis

**Figure 3:**
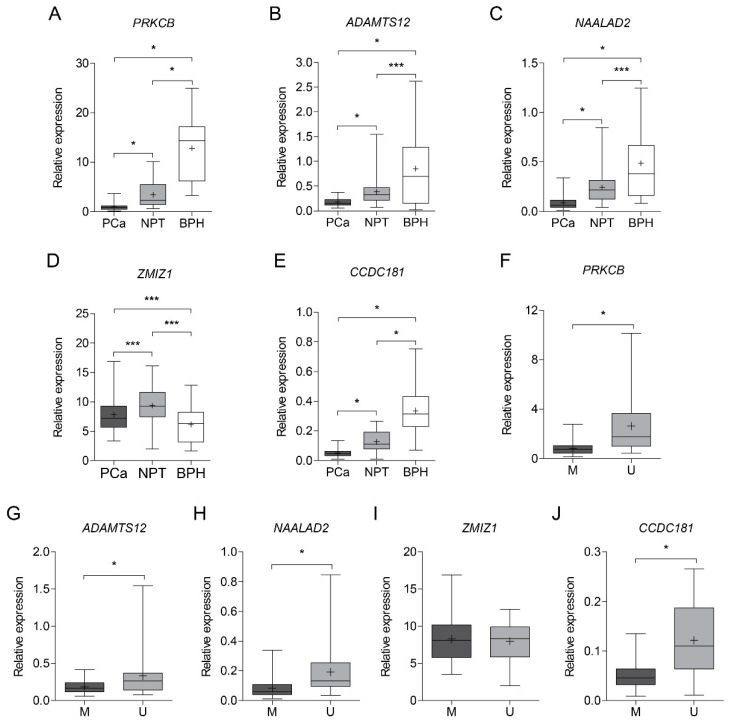
Expression of *ADAMTS12*, *CCDC181*, *NAALAD2*, *PRKCB,* and *ZMIZ1* in cancerous and noncancerous prostate tissue. (**A**–**E**) Relative expression of selected genes in prostate cancer (PCa), noncancerous prostate tissue (NPT), and benign prostatic hyperplasia (BPH) samples; (**F**–**J**) Relative expression of selected genes in PCa according to methylation status, i.e., methylated (M) or unmethylated (U; MSP data). The boxplot depicts Q1–Q3 quartile where the line in the box indicates the median and the plus sign indicates the mean. Significant *p*-values are marked in bold. *—*p* ≤ 0.0001, ***—*p* < 0.05.

Based on promoter methylation frequencies and correlations with clinico-pathological variables, *ADAMTS12*, *CCDC181*, *NAALAD2*, *PRKCB,* and *ZMIZ1* were selected for further expression analysis. RNA with suitable quality for molecular analysis was available from 81 PCa, 25 NPT, and 17 BPH samples from the same patient cohort (Table S1). *ADAMTS12*, *CCDC181*, *NAALAD2,* and *PRKCB* demonstrated significantly lower expression levels in cancerous prostate tissue than NPT and BPH samples (all *p* < 0.0500; Figure 3A–D). The expression level of *ZMIZ1* was also lower in cancerous than in NPT, but higher than in BPH (all *p* < 0.0500; Figure 3E). Furthermore, lower expression levels of *ADAMTS12*, *CCDC181*, *NAALAD2,* and *PRKCB* in cancerous tissue correlated with promoter methylation status (all *p* ≤ 0.0001; Figure 3F–I), while no such association was demonstrated by *ZMIZ1* (Figure 3J).

Consistent with our findings, significantly lower expression levels of *ADAMTS12*, *CCDC181*, *NAALAD2*, as well as *PRKCB* were identified in PCa as compared to NPT in the PRAD cohort (all *p* < 0.0500), except for *ZMIZ1* (*p* > 0.0500; Figure S2). Moreover, *CCDC181*, *PRKCB,* and *ZMIZ1* demonstrated significantly lower expression levels in cancerous prostate tissue with higher methylation intensity (all *p* < 0.0500), while no statistically significant results were observed for *ADAMTS12* and *NAALAD2* (all *p* > 0.0500; Figure S3).

In our cohort, lower expression levels of *CCDC181* and *NAALAD2* significantly correlated with higher postoperative ISUP grade group (*p* = 0.0016 and *p* = 0.0015; Table S5), while the higher expression level of *CCDC181* was specific for tumors harboring *TMPRSS2-ERG* gene fusion (*p* = 0.0136). No associations between the expression level of any gene and clinico-pathological characteristics, such as pT stage, PSA level, and prostate volume, were observed. However, the expression level of *NAALAD2* revealed a positive correlation with patients’ age (RS = 0.27, *p* = 0.0153; Table S5).

### 3.4. Survival Analysis

**Figure 4:**
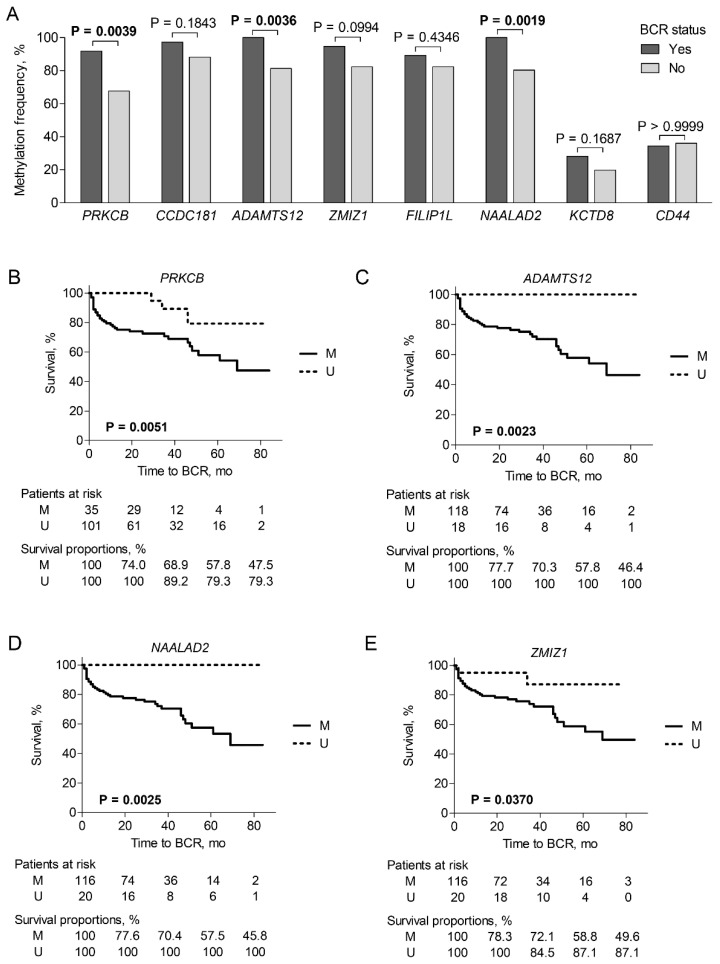
Promoter methylation status of selected genes in prostate cancer (PCa) according to biochemical recurrence (BCR) status. (**A**) Promoter methylation frequencies of the analyzed genes; (**B**–**E**) Kaplan-Meier curves according to methylation of *ADAMTS12*, *NAALAD2*, *PRKCB,* and *ZMIZ1*. M—methylated, U—unmethylated. Significant *p*-values are marked in bold.

BCR-free survival analysis was performed to investigate the prognostic potential of PCa-specific genes. Aberrant promoter methylation of *ADAMTS12*, *NAALAD2* and *PRKCB* was more frequent in patients undergoing BCR than in patients with no biochemical relapse (*p* = 0.0036, *p* = 0.0019 and *p* = 0.0039, respectively; Figure 4A). Comparison of Kaplan-Meier curves revealed significantly lower BCR-free survival rates in PCa patients with aberrant promoter methylation of *ADAMTS12*, *NAALAD2,* and *PRKCB*, as well as *ZMIZ1* (*p* = 0.0023, *p* = 0.0025, *p* = 0.0051 and *p* = 0.0370, respectively; Figure 4B–E). Meanwhile, no statistically significant differences were observed for other investigated biomarkers (not shown).

The prognostic value of *ADAMTS12*, *NAALAD2,* and *PRKCB* was also supported by univariate and multivariate Cox proportional hazard analyses. However, only *NAALAD2* showed significant prognostic value at the gene expression level (all models *p* < 0.0500; Table S6). Forward stepwise variable selection revealed that methylated *NAALAD2* or *PRKCB* together with pT stage or ISUP grade group outperformed the prognostic power of clinico-pathological characteristics alone (all models *p* < 0.0001). Besides, the combination of *PRKCB* promoter methylation status with PSA significantly predicted BCR-free survival, although PSA lacked significance as an independent factor (models *p* = 0.0008 and *p* > 0.0500). Methylation of *ADAMTS12*, *NAALAD2,* and *PRKCB* combined with *TMPRSS2-ERG* fusion also demonstrated a significant prognostic potential for BCR-free survival (all *p* < 0.0500). Selected multivariate Cox models are provided in Table S7.

In TCGA data analysis, PCa cases with ISUP grade group 5, metastatic disease, previous active surveillance, or neoadjuvant therapy were excluded from further analysis to better match our cohort. Similar to our findings, methylation levels of *NAALAD2* and *PRKCB* combined with the pT stage or ISUP grade group revealed significant prognostic value (all models *p* < 0.0500; Table S7). However, in univariate analysis, methylation levels of the selected genes were not associated with BCR status, while decreased expression of *ADAMTS12* and *PRKCB* demonstrated prognostic potential for BCR-free survival (all *p* < 0.0500; Table S6).

## 4. Discussion

Recent progress in genomic technologies, such as microarrays and next-generation sequencing, have opened new possibilities for researchers to conduct genome-wide methylation analysis in various malignancies, primarily focusing on novel biomarkers that could eliminate the limitations of currently used ones. During recent years various studies have made important insight into DNA methylation profile of PCa and identified a large number of novel molecular biomarkers. However, only a few of them have been transferred into clinical practice so far [5,16].

In the current study, PCa methylome data, obtained from well-characterized PCa cohort and matched NPT samples using microarray [10], was applied to screen novel diagnostic and prognostic PCa biomarkers. In agreement with other authors [5,6,16,17], comparing cancerous tissues with NPT and cases with and without BCR, different methylation of various promoters was identified, and the changes affected both intra- and intergenic loci [5,7]. Although microarray design mainly covered various promoter CpG islands regardless of their co-localization with annotated regulatory regions, further validation of selected genes was based on promoter-associated methylation differences.

The majority of the genes reported in our study have been poorly investigated in PCa or other types of malignancies. Some of the most established PCa genes, such as *RARB* and *RASSF1*, were differently methylated in our cohort. However, like APC and GSTP1, others did not show any significant differences in methylation, a phenomenon also reported in other studies [7,16]. Both biological and technical perspectives could at least partially explain all these contradictory results. PCa is a particularly heterogeneous and multifocal disease, leading to differences in biological samples with various cancerous cellularity and aggressiveness. Technical aspects of the procedure, such as different material collection and different processing, storage, research methods, and cut-off interpretation, are also of utmost importance and may have an immense impact on DNA methylation differences between various studies.

Nonetheless, several previously reported genes, such as *CCDC181* [18] and *SEPT9* [19], demonstrated one of the most significant methylation differences in PCa. A 4-gene test, including *CCDC181*, has been proposed to improve PCa detection through epigenetic field effect, where histologically normal prostate tissues in the vicinity of a PCa harbors distinct epigenetic alterations [20]. In agreement, *CCDC181* was the most frequently methylated gene (90.7%) of the 10-gene set in our study, with up to 100% specificity for PCa. In the study by Haldrup et al., methylation of *CCDC181* demonstrated independent value to predict BCR-free survival [18]. However, according to our analysis, the prognostic potential of this gene was not confirmed in our and TCGA cohorts.

The significant role of *ZMIZ1* has been described in PCa progression to a castration-resistant form of the disease through recruitment on the promoter of *KLK3* gene [21]. Similarly, promoter methylation and/or downregulated expression of *FILIP1L* have been associated with an aggressive form of the disease [22,23]. According to our data, no significant differences between *FILIP1L* and *ZMIZ1* methylation frequencies and BCR were observed, which could be explained by the stringent inclusion of low-intermediate risk localized PCa cases into our cohort. However, *ZMIZ1* was more commonly methylated in tumors with advanced pT stage and higher ISUP grade group.

Among the large number of differentially methylated genes identified in the Lithuanian cohort, promoter methylation of *ADAMTS12*, *NAALAD2,* and *PRKCB* had not only high sensitivity (86.1%) and specificity for PCa (≥84.3%) but also revealed potential prognostic value. According to our knowledge, these three genes have never been investigated as DNA methylation biomarkers for PCa.

Methylation of *PRKCB* has been investigated in few types of malignancies, where a positive correlation was identified between methylation of two CpGs and gene expression by analyzing the TCGA dataset of lung adenocarcinoma [24]. In our analysis, *PRKCB* methylation status was strongly associated with decreased gene expression in Lithuanian and TCGA PCa cohorts. Supporting this idea, treatment with demethylating agents has been previously reported to restore *PRKCB* expression [25]. However, this is contradictory data to other studies, reporting up-regulation of *PRKCB* in PCa [26] and its oncogenic role through activated angiogenesis and increased cell proliferation [27]. Furthermore, Metzger et al. have demonstrated that *PRKCB* controls androgen receptor (AR)-dependent gene expression during PCa progression through interaction with *KDM1A* and AR [26]. In our study, promoter methylation of *PRKCB* was predictive of BCR-free survival as an independent factor and in combinations with clinico-pathological characteristics. Because *PRKCB* encodes several distinct isoforms, the role of promoter methylation of *PRKCB* in PCa requires further investigations.

Despite belonging to the same NAALADase gene family as the prostate-specific membrane antigen gene (*PSMA*), little is known about *NAALAD2*. There is a lack of data regarding promoter methylation of the gene. The aberrant methylation of *ADAMTS12*, as a novel tumor suppressor gene, has been previously investigated only in colon cancer, which was associated with disease progression [28]. In our study, promoter methylation of *ADAMTS12* and *NAALAD2* was predictive of BCR-free survival independently or in combination with clinico-pathological characteristics. Besides, together with *PRKCB*, these potential epigenetic biomarkers composed a multivariate model of molecular-only covariates for the prognosis of BCR-free survival. Therefore, the 3-gene methylation assay could provide valuable diagnostic information about the aggressive form of the disease and guide to radical treatment options to obtain the best possible oncological outcomes and assist in BCR prognosis making that could lead to early adjuvant or salvage treatment options. However, further investigations in independent cohorts are needed to confirm our findings before implementing these specific biomolecular markers into clinics.

## 5. Conclusions

Three novel PCa-specific biomarkers were identified through genome-wide screening of DNA methylation changes in PCa. Promoter methylation of *ADAMTS12*, *NAALAD2,* and *PRKCB* showed high sensitivity and specificity for PCa. The methylation status of investigated genes was also independently predictive for poor BCR-free survival and had even higher prognostic power in models with the established clinico-pathological characteristics. Future studies in larger cohorts are needed to further assess the proposed biomarker assay’s clinical value and evaluate its potential for noninvasive testing in body fluids, like urine or blood.

## 6. Patents

Resulting to this work, a patent application “Characterization of Prostate Cancer Using DNA Methylation Assay System” No. PCT/IB2019/056204 was submitted on 18 September 2019, which latter was licensed to “ThermoPharma Baltic”.

## Data Availability

All data supporting the results reported in the article is available from the corresponding author upon a reasonable request.

## Supplementary Materials

The following are available online at https://www.mdpi.com/article/10.3390/ijms22116091/s1.
