# Supplementary material for: Promoter Methylation of PRKCB, ADAMTS12, and NAALAD2 Is Specific to Prostate Cancer and Predicts Biochemical Disease Recurrence
Source: Int J Mol Sci. 2021 Jun 5;22(11):6091. doi: 10.3390/ijms22116091 (PMC8201120; doi:10.3390/ijms22116091)
Supplement: Supplementary file 1 [file ijms-22-06091-s001.zip › ijms-1149760 Supplementary materials.pdf]

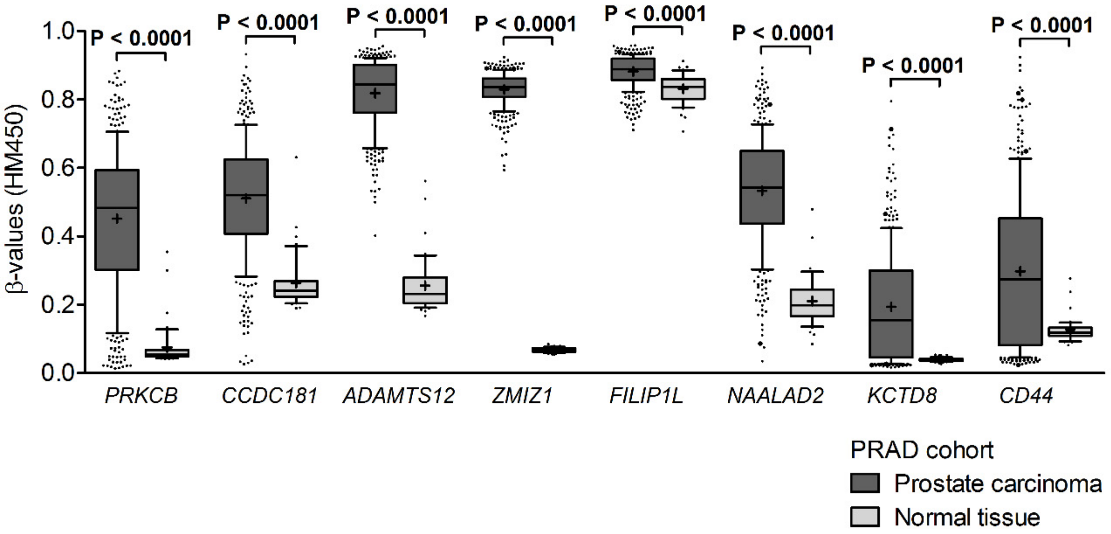


**Figure S1.** DNA methylation levels of selected genes in the Prostate Cancer Dataset (PRAD) [9]. Level 3 DNA methylation data, obtained using HM450 platform, were used to generate the plots. The depicted β-values are average values of the gene promoter regions. The boxplot depicts Q1-Q3 quartile, when the line in the box indicates the median, the plus sign indicates the mean, the whiskers represent 10-90% of the range and the outliers are marked as dots. Significant P-values are marked in bold.

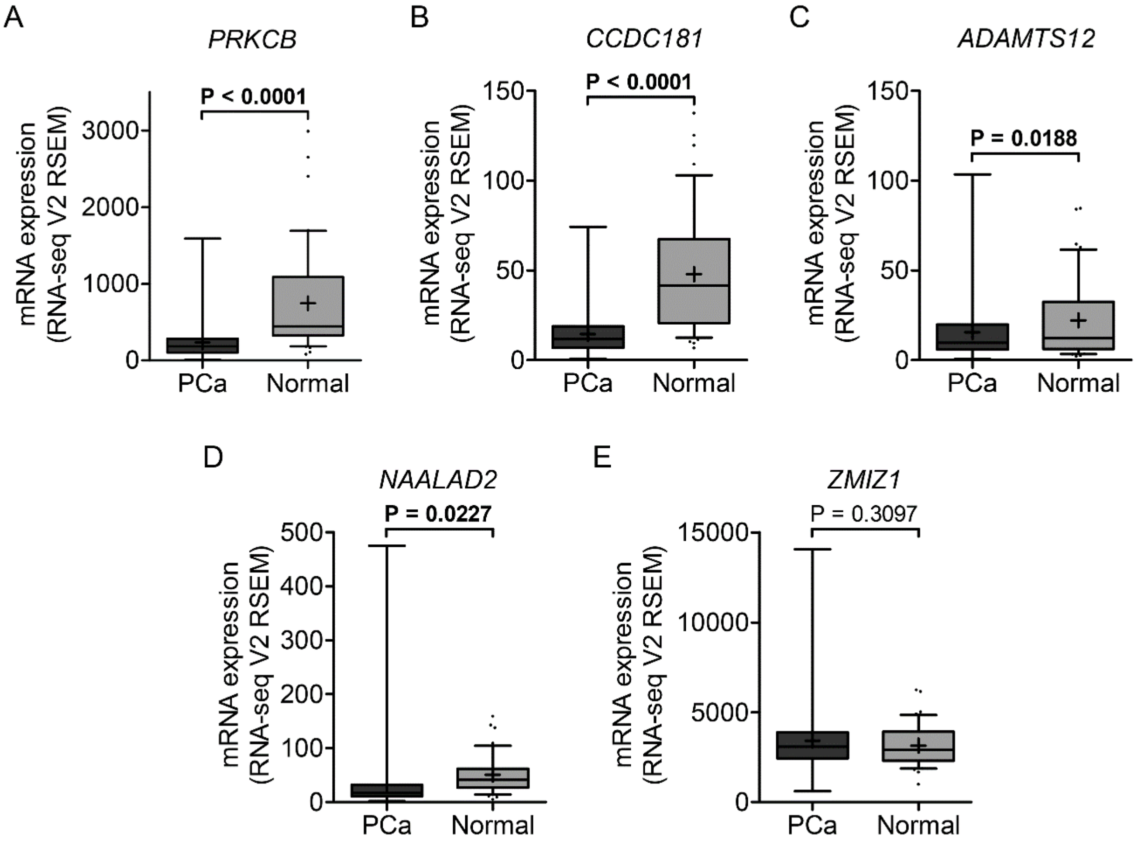


**Figure S2.** Relative expression levels of selected genes in the Prostate Cancer Dataset (PRAD) [9]. Level 3 PRAD RNA-seq RSEM data were used to generate the plots. The boxplot depicts Q1-Q3 quartile, when the line in the box indicates the median, the plus sign indicates the mean, the whiskers represent 10-90% of the range and the outliers are marked as dots. Significant P-values are marked in bold.

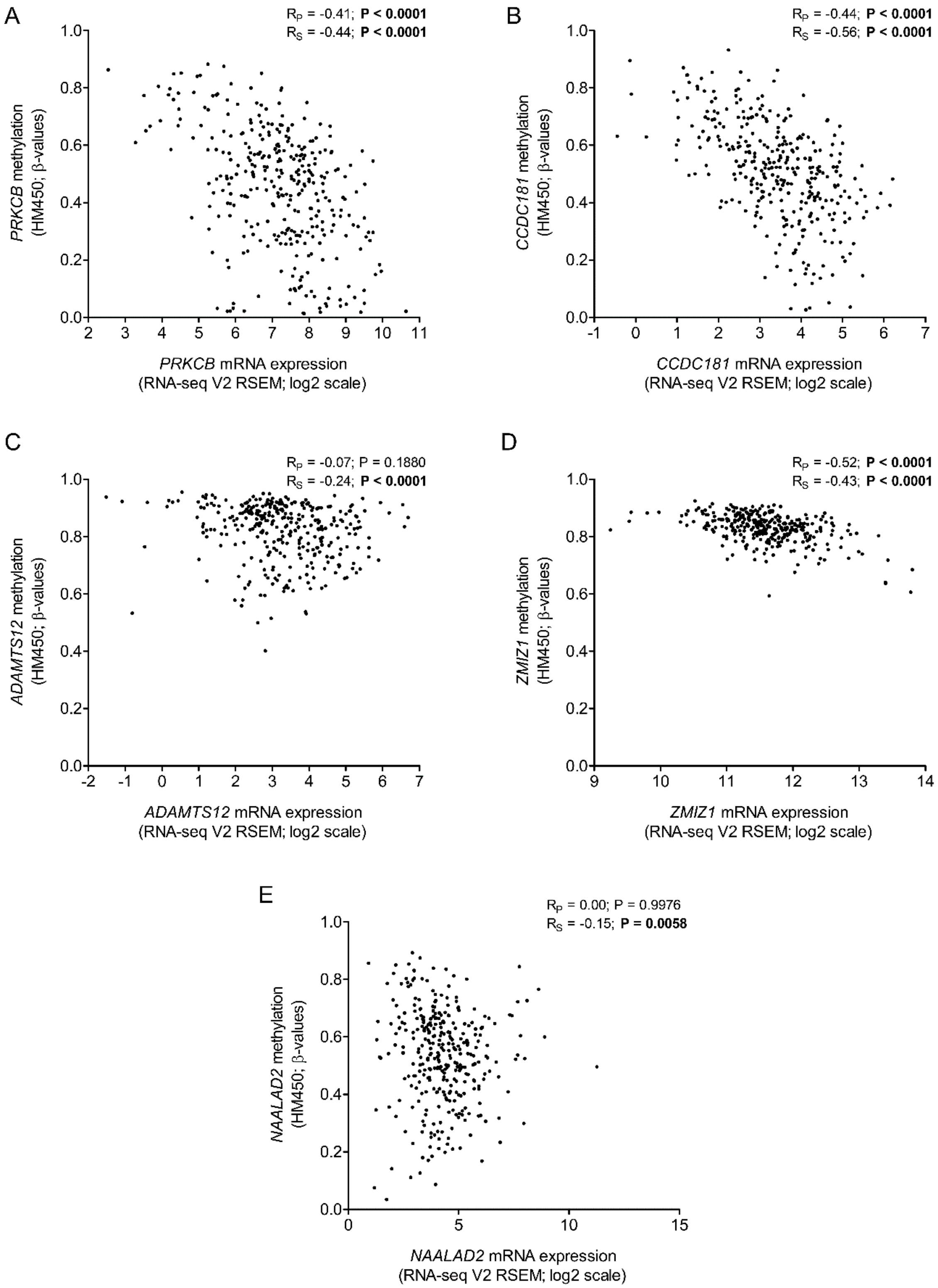


**Figure S3.** Correlations between promoter methylation status and gene expression levels of selected genes in the Prostate Cancer Dataset (PRAD) [9]. Level 3 DNA methylation data, obtained using HM450 platform, and level 3 PRAD RNA-seq RSEM data were used to generate scatter diagrams. RNA-seq data is plotted on log2 scale. Pearson's R (RP) and Spearman's R (RS) correlation coefficients are provided in the graphs. Significant P-values are marked in bold.

**Table S1.** Clinico-pathological and molecular characteristics of the analysis groups.

| Parameter | Methylation Analysis Group | | | Gene Expression Analysis Group [1] | |
|---|---|---|---|---|---|
| **Group composition** | **PCa (N = 151)** | **NPT (N = 51)** | **BPH (N = 17)** | **PCa (N = 81)** | **NPT (N = 25)** |
| Tumor stage, N | | | | | |
| ≤pT2 | 96 | - | - | 51 | - |
| ≥pT3 | 55 | - | - | 30 | - |
| ISUP grade group (Gleason score), N | | | | | |
| I (3 + 3) | 34 | - | - | 13 | - |
| II (3 + 4) | 84 | - | - | 48 | - |
| III (4 + 3) | 23 | - | - | 14 | - |
| IV (8) | 3 | - | - | 2 | - |
| V (9) | 5 | - | - | 3 | - |
| Unknown | 2 | - | - | 1 | - |
| Tumor cellularity, N | | | | | |
| 90-100% | 73 | - | - | 49 | - |
| 70-89% | 38 | - | - | 24 | - |
| 50-69% | 40 | - | - | 8 | - |
| 1-3% | - | 3 | - | - | 0 |
| 0% | - | 48 | - | - | 25 |
| BCR status, N | | | | | |
| Yes (mean time to BCR, mo) | 37 (18) | 16 (16) | - | 30 (19) | 7 (20) |
| No (mean follow-up, mo) | 99 (38) | 32 (47) | - | 49 (42) | 17 (58) |
| Unknown | 15 | 3 | - | 2 | 1 |
| PSA, ng/mL | | | | | |
| Mean±SD (range) | 10.6±11.1 [2.5; 84.2] | 9.4±8.6 [2.5; 44.0] | 7.3±6.6 [0.8; 28.1] | 10.8±9.4 [2.5; 44.0] | 10.0±9.5 [2.5; 44.0] |
| Unknown | 2 | 1 | 0 | 2 | 1 |
| Prostate mass, g | | | | | |
| Mean±SD (range) | 48±17 [16; 123] | 50±20 [16; 104] | - | 48±17 [16; 123] | 48±20 [16; 104] |
| *TMPRSS2-ERG* fusion transcript, N | | | | | |
| Yes | 77 | - | - | 46 | - |
| No | 44 | - | - | 26 | - |
| Unknown | 8 | - | - | 9 | - |
| Age, years | | | | | |
| Mean±SD (range) | 61±8 [41; 82] | 62±7 [46; 74] | 70±8 [59; 80] | 61±8 [41;82] | 61±6 [48; 74] |

BCR—biochemical disease recurrence, PCa—prostate cancer, NPT—noncancerous prostate tissue, BPH—benign prostatic hyperplasia, PSA—prostate-specific antigen, SD—standard deviation. [1] The same subset of BPH samples as in "Methylation analysis in group" was also included in the gene expression analysis.

**Table S2.** Primers used for methylation-specific PCR, location of amplicons, and amplification conditions.

| Gene symbol | Primer pair ID | Forward primer sequence (5'-3') | Reverse primer sequence (5′-3′) | Product size, nt | Amplicon location from TSS | Primer annealing T, °C | Number of PCR cycles |
|---|---|---|---|---|---|---|---|
| *PRKCB* | M | TAAGCGTAGTTGGACGAGC | AAAACGACGACCGCTACTAC | 124 | -7/+117 | 56 | 36 |
| | U | TGTTAAGTGTAGTTGGATGAGT | AAAACAACAACCACTACTACACC | 127 | -10/+117 | | |
| *CCDC181* (*C1orf114*) | M | CGGTATTTCGCGAGTTTTTATAAC | CGAAAACGACAAAAATCTACG | 164 | -166/-3 | 57 | 35 |
| | U | TAGTGGTATTTTGTGAG-TTTTTATAAT | ACAAAAACAACAAAAATCTACACA | 168 | -169/-2 | | |
| *ADAMTS12* | M | GAGTTCGGGAGGAAGATGTATC | ACAACGACTACAAAACTACCCG | 241 | -195/+46 | 62 | 35 |
| | U | GAGTTTGGGAGGAAGATGTATT | AAACAACAACTACAAAACTACCA | 243 | -195/+48 | | |
| *NAALAD2* | M | TATTTATTATGTTCGGGTTATTGC | CCTACTACATTCGCGAACTTC | 244 | -116/+128 | 58 | 35 |
| | U | GTTATTTATTATGTTTGGGTTATTGT | CCTACTACATTCACAAACTTCAA | 246 | -118/+128 | | |
| *ZMIZ1* | M | TCGTTTCGAAAATTTTTTAAATC | AACTCCCGAAACGCTATC | 246 | -188/+58 | 55 | 38 |
| | U | TGTAGTTTGTTTT-GAAAATTTTTTAAA | AACTCCCAAAACACTATCACC | 252 | -194/+58 | | |
| *FILIP1L* | M | TACGGTTCGTTTATACGGTC | CGACCTATAAACGTTACGTCA | 160 | -151/+9 | 57 | 36 |
| | U | GGAATTATGGTTTGTTTATATGGTT | CCCAACCTATAAACATTACATCAC | 167 | -156/+11 | | |
| *KCTD8* | M | TTTTTATTGTCGTCGTCGTATC | CTCCGCGTACTCCTAACG | 169 | +45/+213 | 58 | 37 |
| | U | GTTTTTTTATTGTTGTTGTTGTATT | ACCCTCCACATACTCCTAACA | 175 | +42/+216 | | |
| *CD44* | M | TCGTTGAGTTTGGCGTAGATC | ACTACCGCCGAATCCGCG | 89 | +480/+568 | 58 | 35 |
| | U | GTGTTGTTGAGTTTGGTGTAGATT | CAAAAAAACTACCAC-CAAATCCACA | 99 | +477/+575 | | |
| *EPAS1* | M | ATATATTCGCGTCGGTGTTC | CGCTCGCGAATATAAAACTC | 118 | -100/+18 | 56-57 | 37-38 |
| | U | TGTTTATATATTTGTGTTGGTGTTTG | CCACTCACAAATATAAAACTCCC | 124 | -105/+19 | | |
| *NEK9* | M | TTAATAGATTCGAGAGGTCGTATC | CGCGAAACTCGATAATAACTC | 253 | -210/+43 | 57 | 38 |
| | U | GGGTTAATAGATTTGAGAGGTT-GTATT | CCACAAAACTCAATAATAACTCCT | 257 | -213/+44 | | |

All primers were designed with Methyl Primer Express® Software v1.0 (Applied Biosystems™, Thermo Fisher Scientific, Carlsbad, CA, USA). TSS – transcription start site, M/ U – primers specific for methylated/ unmethylated sequence.

**Table S3.** Primers used for quantitative methylation-specific PCR and location of amplicons.

| Gene symbol | Primer ID | Primer sequence (5'-3') | Amplicon size, bp | Amplicon location from TSS | Reference |
|---|---|---|---|---|---|
| *PRKCB* | Fw | CGTAGTTGGGGTTAGCGGTG | 145 | -28/+117 | This study |
| | Rv | AAAACGACGACCGCTACTACA | | | |
| | Probe | JOE-TTAGAGTCGGCGTAGGGGAAGCG-BHQ-1 | | | |
| *CCDC181* (*C1orf114*) | Fw | GCGGTATTTCGCGAGTTTTTAT | 131 | -167/-37 | This study |
| | Rv | TATCCTCAAACCACCGACC | | | |
| | Probe | FAM-AGTATCGGGATGGGTGTCGGGA-BHQ-1 | | | |
| *ADAMTS12* | Fw | CGGGAGGAAGATGTATCGAGC | 138 | -190/-53 | This study |
| | Rv | TCAACTAACAATATCCGCTTTCG | | | |
| | Probe | Cy5-TTTCGTTTTGGTTTATTTTATATTTCG-BHQ-3 | | | |
| *NAALAD2* | Fw | TGCGAAGGTTAGCGGAGGT | 139 | +36/+174 | This study |
| | Rv | GACCCCTAAATTCCGCCATAA | | | |
| | Probe | FAM-GAAGTTCGCGAATGTAGTAGGCG-BHQ-1 | | | |
| *ACTB* | Fw | TGGTGATGGAGGAGGTTTAGTAAGT | 133 | -1629/-1497 | [29] |
| | Rv | AACCAATAAAACCTACTCCTCCCTTAA | | | |
| | Probe | FAM-ACCACCACCCAACACACAATAACAAACAC A-BHQ-1 | | | |

TSS – transcription start site, Fw/ Rv – forward/ reverse primer.

Ref [29]. Lehmann, U.; Langer, F.; Feist, H.; Glöckner, S.; Hasemeier, B.; Kreipe, H. Quantitative assessment of promoter hypermethylation during breast cancer de-velopment. *Am. J. Pathol.* **2002**, *160*, 605–612.

**Table S4.** Genes showing the most significant differences of methylation levels according to tissue histology and biochemical disease recurrence status.

| PCa vs. NPT | | | | | | BCR-positive vs. BCR-negative | | | | | |
|---|---|---|---|---|---|---|---|---|---|---|---|
| **No.** | **Gene/ locus** | **Probe location** | **Methy-lation** | **FC** | **P-value** | **No.** | **Gene/ locus** | **Probe location** | **Methy-lation** | **FC** | **P-value** |
| 1 | *HOXC4* | Intragenic | up | 1.3 | <0.0001 | 1 | *COL23A1* | Intragenic | up | 1.3 | <0.0001 |
| 2 | *CCDC181* | Promoter | up | 1.4 | <0.0001 | 2 | *HOXC9* | Promoter | up | 1.2 | <0.0001 |
| 3 | *MAGI2* | Intragenic | up | 1.5 | <0.0001 | 3 | *MATN4* | Promoter | up | 1.2 | <0.0001 |
| 4 | *CHGB* | Promoter | up | 1.2 | <0.0001 | 4 | *POU4F3* | Intragenic | up | 1.3 | <0.0001 |
| 5 | *C8orf33* | Promoter | down | 1.4 | <0.0001 | 5 | *MECOM* | Promoter | down | 1.2 | <0.0001 |
| 6 | *SOX2OT* | Intragenic | up | 1.4 | <0.0001 | 6 | *CAMTA1* | Intragenic | up | 1.4 | <0.0001 |
| 7 | *PNMA6A* | Intragenic | down | 1.6 | <0.0001 | 7 | *C7orf51* | Intragenic | up | 1.3 | 0.0001 |
| 8 | *CHAT* | Promoter | up | 1.4 | <0.0001 | 8 | *BMP8A* | Intragenic | up | 1.2 | 0.0001 |
| 9 | *ARMC9* | Intragenic | down | 1.2 | <0.0001 | 9 | *RAPGEFL1* | Promoter | up | 1.3 | 0.0001 |
| 10 | *DGKG* | Intragenic | up | 1.3 | <0.0001 | 10 | *BARHL1* | Promoter | up | 1.2 | 0.0001 |
| 11 | *FER1L4* | Intragenic | up | 1.3 | <0.0001 | 11 | *C1orf170* | Promoter | up | 1.5 | 0.0001 |
| 12 | *SLC32A1* | Intragenic | up | 1.4 | <0.0001 | 12 | *TMEM233* | Intragenic | up | 1.2 | 0.0001 |
| 13 | *PCDHGC4* | Intragenic | up | 1.3 | <0.0001 | 13 | *C1D* | Intragenic | down | 1.2 | 0.0002 |
| 14 | *DPYSL3* | Promoter | up | 1.3 | <0.0001 | 14 | *NFATC1* | Promoter | down | 1.3 | 0.0002 |
| 15 | *GRIA3* | Promoter | up | 1.3 | <0.0001 | 15 | *CLDN5* | Intragenic | up | 1.3 | 0.0002 |
| 16 | *GATA4* | Intragenic | up | 1.2 | <0.0001 | 16 | *PLK1* | Intragenic | up | 1.3 | 0.0002 |
| 17 | *SGMS1* | Intragenic | up | 1.4 | <0.0001 | 17 | *CACNG7* | Intragenic | up | 1.3 | 0.0002 |
| 18 | *ZIC1* | Intragenic | up | 1.2 | <0.0001 | 18 | *GSX2* | Intragenic | up | 1.2 | 0.0002 |
| 19 | *C1orf229* | Promoter | down | 1.3 | <0.0001 | 19 | *C19orf20* | Intragenic | up | 1.3 | 0.0002 |
| 20 | *CXCL6* | Intragenic | up | 1.4 | <0.0001 | 20 | *EPHA10* | Intragenic | up | 1.3 | 0.0002 |
| 21 | *DRGX* | Promoter | up | 1.4 | <0.0001 | 21 | *SPRY4* | Intragenic | down | 1.2 | 0.0002 |
| 22 | *LPPR4* | Intragenic | up | 1.3 | <0.0001 | 22 | *WNT10B* | Intragenic | up | 1.3 | 0.0002 |
| 23 | *MKNK1* | Promoter | up | 1.2 | <0.0001 | 23 | *TRIM54* | Intragenic | up | 1.2 | 0.0002 |
| 24 | *NR2E1* | Intragenic | up | 1.4 | <0.0001 | 24 | *SV2A* | Intragenic | up | 1.4 | 0.0003 |
| 25 | *MCF2L2* | Intragenic | up | 1.4 | <0.0001 | 25 | *HOPX* | Intragenic | up | 1.2 | 0.0003 |
| 26 | *DPP10* | Intragenic | up | 1.4 | <0.0001 | 26 | *RSPH9* | Intragenic | up | 1.3 | 0.0003 |
| 27 | *SLC24A2* | Intragenic | up | 1.3 | <0.0001 | 27 | *HOXB4* | Intragenic | up | 1.3 | 0.0003 |
| 28 | *LOC648809* | Promoter | up | 1.2 | <0.0001 | 28 | *SYP* | Intragenic | up | 1.2 | 0.0003 |
| 29 | *ALDH1L2* | Intragenic | up | 1.3 | <0.0001 | 29 | *C2orf88* | Promoter | up | 1.2 | 0.0003 |
| 30 | *HOXC11* | Intragenic | up | 1.3 | <0.0001 | 30 | *CALY* | Intragenic | up | 1.3 | 0.0003 |
| 31 | *NAALAD2* | Promoter | up | 1.5 | <0.0001 | 31 | *FAM43B* | Intragenic | up | 1.2 | 0.0003 |
| 32 | *EZH2* | Promoter | down | 1.4 | <0.0001 | 32 | *IGFBP7* | Intragenic | up | 1.4 | 0.0003 |
| 33 | *TTC23L* | Intragenic | up | 1.2 | <0.0001 | 33 | *PLCXD1* | Promoter | down | 1.3 | 0.0004 |

| | | | | | | | | | | | |
|---|---|---|---|---|---|---|---|---|---|---|---|
| 34 | *SPAG17* | Promoter | up | 1.4 | <0.0001 | 34 | *WBP2* | Promoter | down | 1.2 | 0.0004 |
| 35 | *FGD5* | Promoter | up | 1.6 | <0.0001 | 35 | *SCNN1B* | Promoter | up | 1.2 | 0.0004 |
| 36 | *GALNT13* | Intragenic | up | 1.2 | <0.0001 | 36 | *LOC400043* | Intragenic | up | 1.2 | 0.0004 |
| 37 | *ZSCAN12* | Intragenic | up | 1.3 | <0.0001 | 37 | *ZNF778* | Intragenic | up | 1.2 | 0.0004 |
| 38 | *TRIM7* | Intragenic | up | 1.3 | <0.0001 | 38 | *MMP25* | Intragenic | up | 1.3 | 0.0004 |
| 39 | *CNPY1* | Intragenic | up | 1.3 | <0.0001 | 39 | *LOC100287042* | Intragenic | down | 1.3 | 0.0004 |
| 40 | *HOXC10* | Intragenic | up | 1.4 | <0.0001 | 40 | *NRXN2* | Intragenic | up | 1.4 | 0.0005 |
| 41 | *GPR149* | Intragenic | up | 1.4 | <0.0001 | 41 | *EXOC3L2* | Intragenic | up | 1.3 | 0.0005 |
| 42 | *PAX7* | Intragenic | up | 1.3 | <0.0001 | 42 | *INSM1* | Promoter | up | 1.3 | 0.0005 |
| 43 | *DLL4* | Promoter | up | 1.3 | <0.0001 | 43 | *SEPT9* | Promoter | up | 1.3 | 0.0005 |
| 44 | *BHMT* | Intragenic | up | 1.3 | <0.0001 | 44 | *C1orf190* | Promoter | up | 1.2 | 0.0005 |
| 45 | *C6orf97* | Promoter | up | 1.2 | <0.0001 | 45 | *SPOCK2* | Intragenic | up | 1.2 | 0.0005 |
| 46 | *CA3* | Promoter | up | 1.4 | <0.0001 | 46 | *ALLC* | Promoter | up | 1.6 | 0.0005 |
| 47 | *TFPI2* | Promoter | up | 1.2 | <0.0001 | 47 | *MIR152* | Promoter | up | 1.4 | 0.0005 |
| 48 | *VSX1* | Promoter | up | 1.2 | <0.0001 | 48 | *FAM100A* | Promoter | down | 1.2 | 0.0005 |
| 49 | *C4orf22* | Intragenic | up | 1.2 | <0.0001 | 49 | *SPTBN4* | Promoter | up | 1.3 | 0.0005 |
| 50 | *HELT* | Intragenic | up | 1.2 | <0.0001 | 50 | *ZNF471* | Promoter | down | 1.3 | 0.0005 |

Values are given for the most significant microarray probe per gene/ annotated locus. PCa – prostate tumors, NPT – noncancerous prostate tissues, BCR – biochemical disease recurrence, FC – absolute fold change value.

**Table S5.** Associations of promoter methylation and gene expression with clinico-pathological variables and *TMPRSS2-ERG* fusion status in prostate tumors.

| **Promoter methylation** | **pT3-4 vs. pT2** | | **pISUP grade group** | | ***TMPRSS2-ERG*+ vs. -** | | **PSA** | | **Prostate volume** | | **Age** | |
|---|---|---|---|---|---|---|---|---|---|---|---|---|
| | **%** | **P-value** | **H** | **P-value** | **%** | **P-value** | **$Z_{ad}$** | **P-value** | **$Z_{ad}$** | **P-value** | **$Z_{ad}$** | **P-value** |
| *PRKCB* | 81.8 vs. 66.7 | 0.0589 | 7.19 | 0.0659 | 83.1 vs. 61.4 | **0.0151** | 1.91 | 0.0555 | -0.17 | 0.4433 | 0.11 | 0.9123 |
| *CCDC181* | 94.5 vs. 88.5 | 0.2594 | 2.85 | 0.4150 | 92.2 vs. 88.6 | 0.5260 | 0.22 | 0.8275 | 1.22 | 0.2237 | 1.30 | 0.1924 |
| *ADAMTS12* | 92.7 vs. 82.3 | 0.0898 | 5.58 | 0.1338 | 89.6 vs. 75.0 | **0.0406** | 0.43 | 0.6665 | -0.70 | 0.4859 | 1.28 | 0.2021 |
| *ZMIZ1* | 94.5 vs. 81.3 | **0.0273** | 6.46 | 0.0914 | 85.7 vs. 86.4 | >0.9999 | 1.94 | 0.0528 | 0.58 | 0.5593 | 1.41 | 0.1577 |
| *FILIP1L* | 89.1 vs. 78.1 | 0.1223 | 1.93 | 0.5875 | 88.3 vs. 75.0 | 0.0755 | 0.70 | 0.4810 | 0.70 | 0.4810 | -0.12 | 0.9071 |
| *NAALAD2* | 74.5 vs. 65.6 | 0.2785 | 7.57 | 0.0557 | 71.4 vs. 70.5 | >0.9999 | 1.60 | 0.1105 | -0.06 | 0.9487 | 0.76 | 0.4483 |
| *CD44* | 36.4 vs. 33.3 | 0.7248 | 0.60 | 0.8957 | 40.3 vs. 20.5 | **0.0286** | -0.43 | 0.6686 | 0.91 | 0.3612 | 1.12 | 0.2639 |
| *KCTD8* | 36.4 vs. 12.5 | **0.0008** | 6.49 | 0.0902 | 31.2 vs. 6.8 | **0.0015** | 0.98 | 0.3248 | -0.49 | 0.6275 | 0.44 | 0.6568 |

| Gene expression | pT | | pISUP grade group | | *TMPRSS2-ERG*+ vs. - | | PSA | | Prostate volume | | Age | |
|---|---|---|---|---|---|---|---|---|---|---|---|---|
| | $Z_{ad}$ | P-value | $R_S$ | P-value | $Z_{ad}$ | P-value | $R_S$ | P-value | $R_S$ | P-value | $R_S$ | P-value |
| *PRKCB* | -0.45 | 0.6528 | -0.16 | 0.1439 | 0.17 | 0.8674 | -0.17 | 0.1362 | 0.00 | 0.9815 | 0.13 | 0.2456 |
| *CCDC181* | -0.65 | 0.5154 | -0.35 | **0.0016** | 2.47 | **0.0136** | -0.20 | 0.0755 | -0.01 | 0.9482 | 0.15 | 0.1698 |
| *ADAMTS12* | -0.35 | 0.7248 | 0.13 | 0.2570 | 0.97 | 0.3323 | -0.12 | 0.2958 | 0.05 | 0.6676 | 0.01 | 0.9334 |
| *ZMIZ1* | -0.29 | 0.7692 | -0.07 | 0.5211 | -0.09 | 0.9313 | 0.11 | 0.3433 | 0.03 | 0.8052 | 0.02 | 0.8385 |
| *NAALAD2* | -1.07 | 0.2864 | -0.35 | **0.0015** | -0.03 | 0.9742 | -0.19 | 0.0972 | -0.03 | 0.8003 | 0.27 | **0.0153** |

pT – pathological tumor stage, pISUP – pathological ISUP grading, ISUP – International Society of Urological Pathology, *TMPRSS2-ERG*+/ - – *TMPRSS2-ERG* fusion positive/ negative status, PSA – prostate-specific antigen, H – Kruskal-Wallis's H parameter, $Z_{ad}$ – Mann-Whitney's Z adjusted parameter, $R_S$ – Spearman's correlation coefficient. Significant P-values are in bold.

**Table S6.** Univariate Cox proportional hazard analysis of molecular and/or clinico-pathological variables in Lithuanian and The Cancer Genome Atlas (TCGA) prostate cancer cohorts. For simplicity, only models' P-values are provided for TCGA data.

| No. | Covariates | Lithuanian cohort | | | TCGA cohort | |
|---|---|---|---|---|---|---|
| | | **HR (95% CI)** | **Covariate's P-value** | **Model's P-value** | **HR (95% CI)** | **Model's P-value** |
| Clinico-pathological variables | | | | | | |
| 1 | pT (3 *vs.* 2) | 4.68 (2.30; 9.51) | **<0.0001** | **<0.0001** | 0.11 (0.03; 0.49) | **0.0001** |
| 2 | pISUP grade group (5 groups) | 2.93 (2.06; 4.16) | **<0.0001** | **<0.0001** | 2.00 (1.44; 2.80) | **<0.0001** |
| 3 | Prostate volume (cont.) | 1.01 (0.99; 1.03) | 0.2001 | 0.2226 | n. a. | n. a. |
| 4 | PSA (cont.) | 1.02 (1.00; 1.04) | **0.0342** | 0.0682 | 1.02 (0.96; 1.08) | 0.4881 |
| 5 | Age (cont.) | 1.00 (0.95; 1.04) | 0.9241 | 0.9239 | 1.02 (0.96; 1.08) | 0.4655 |
| Promoter methylation | | | | | | |
| 6 | *PRKCB* (M *vs.* U/ cont.) | 6.31 (1.52; 26.10) | **0.0115** | **0.0008** | 4.90 (0.81; 29.59) | 0.0795 |
| 7 | *CCDC181* (M *vs.* U/ cont.) | 4.18 (0.58; 30.29) | 0.1585 | 0.0725 | 7.22 (0.85; 61.18) | 0.0702 |
| 8 | *ADAMTS12* (M *vs.* U/ cont.) | >1000 | 0.9496 | **0.0003** | 49.36 (0.60; 4093.06) | 0.0624 |
| 9 | *NAALAD2* (M *vs.* U/ cont.) | 6.08 (1.86; 19.84) | **0.0029** | **0.0002** | 7.38 (0.69; 79.12) | 0.0911 |
| 10 | *ZMIZ1* (M *vs.* U/ cont.) | 3.51 (0.85; 14.57) | 0.0845 | **0.0368** | 0.28 (0.00; 107.98) | 0.6455 |
| Gene expression | | | | | | |
| 11 | *PRKCB* (cont.) | 0.60 (0.27; 1.34) | 0.2118 | 0.1836 | 1.02 (1.00; 1.04) | **0.0378** |
| 12 | *CCDC181* (cont.) | 0.00 (0.00; 28.14) | 0.1329 | 0.1111 | 0.98 (0.94; 1.01) | 0.1856 |
| 13 | *ADAMTS12* (cont.) | 2.26 (0.02; 262.82) | 0.7375 | 0.7387 | 1.02 (1.00; 1.04) | **0.0321** |
| 14 | *ZMIZ1* (cont.) | 0.94 (0.82; 1.09) | 0.4090 | 0.3960 | 1.00 (1.00; 1.00) | 0.0548 |
| 15 | *NAALAD2* (cont.) | 0.00 (0.00; 0.17) | **0.0205** | **0.0059** | 1.00 (0.99; 1.00) | 0.7077 |
| 16 | *TMPRSS2-ERG* (yes *vs.* no) | 0.70 (0.34; 1.44) | 0.3328 | 0.3375 | n. a. | n. a. |

M/U – methylated/ unmethylated promoter status, cont. – continuous variable, *TMPRSS2-ERG* – fusion transcript status, pT – pathological tumor stage, pISUP – pathological ISUP grading, ISUP – International Society of Urological Pathology, PSA – prostate-specific antigen, HR – hazard ratio, CI – confidence intervals. Significant P-values are in bold, n.a. – not available/ not analyzed.

**Table S7.** Multivariate Cox proportional hazard analysis of protein-coding gene methylation and other molecular and/or clinico-pathological variables in Lithuanian and The Cancer Genome Atlas (TCGA) prostate cancer cohorts.

| No. | Covariates | Lithuanian cohort | | | TCGA cohort |
|---|---|---|---|---|---|
| | | HR (95% CI) | Covariate's P-value | Model's P-value | Model's P-value |
| Combinations with clinico-pathological variables | | | | | |
| 1 | pT (3 *vs.* 2) | 4.14 (2.04; 8.42) | **0.0001** | **<0.0001*** | **0.0002** |
| | *PRKCB* (M *vs.* U/ cont.) | 5.18 (1.24; 21.55) | **0.0245** | | |
| 2 | pT (3 *vs.* 2) | 4.34 (2.14; 8.82) | **0.0001** | **<0.0001*** | **0.0001** |
| | *NAALAD2* (M *vs.* U/ cont.) | 5.55 (1.70; 18.15) | **0.0048** | | |
| 3 | pISUP grade group (5 groups) | 2.65 (1.86; 3.79) | **<0.0001** | **<0.0001*** | **<0.0001** |
| | *PRKCB* (M *vs.* U/ cont.) | 4.51 (1.07; 18.93) | **0.0408** | | |
| 4 | pISUP grade group (5 groups) | 2.61 (1.83; 3.72) | **<0.0001** | **<0.0001*** | **<0.0001** |
| | *NAALAD2* (M *vs.* U/ cont.) | 4.50 (1.36; 14.94) | **0.0145** | | |
| 5 | PSA (cont.) | 1.02 (1.00; 1.04) | **0.0403** | **0.0008*** | 0.5903 |
| | *PRKCB* (M *vs.* U/ cont.) | 6.28 (1.52; 26.02) | **0.0117** | | |
| 6 | PSA (cont.) | 1.02 (1.00; 1.04) | 0.0862 | **0.0001** | 0.7838 |
| | *PRKCB* (M *vs.* U/ cont.) | 3.76 (0.87; 16.25) | 0.0778 | | |
| | *NAALAD2* (M *vs.* U/ cont.) | 3.70 (1.09; 12.60) | **0.0371** | | |
| Combinations of gene promoter methylation | | | | | |
| 7 | *PRKCB* (M *vs.* U/ cont.) | 3.84 (0.90; 16.35) | 0.0704 | **0.0001** | 0.1314 |
| | *NAALAD2* (M *vs.* U/ cont.) | 4.30 (1.28; 14.46) | **0.0191** | | |
| 8 | *ADAMTS12* (M *vs.* U/ cont.) | >1000 | 0.9522 | **<0.0001** | 0.1132 |
| | *NAALAD2* (M *vs.* U/ cont.) | 3.81 (1.16; 12.46) | **0.0279** | | |
| 9 | *PRKCB* (M *vs.* U/ cont.) | 3.28 (0.79; 13.63) | 0.1033 | **<0.0001** | 0.2001 |
| | *ADAMTS12* (M *vs.* U/ cont.) | >1000 | 0.9524 | | |
| | *NAALAD2* (M *vs.* U/ cont.) | 3.46 (1.04; 11.45) | **0.0433** | | |
| 10 | *NAALAD2* (M *vs.* U/ cont.) | 5.10 (1.18; 22.08) | **0.0303** | **0.0007** | 0.2267 |
| | *NAALAD2* (expression, cont.) | 0.00 (0.00; 2.70) | 0.0828 | | |
| Combinations with fusion transcript status | | | | | |
| 11 | *TMPRSS2-ERG* (yes *vs.* no) | 1.61 (0.80; 3.27) | 0.1872 | **0.0113** | n.a. |
| | *PRKCB* (M *vs.* U/ cont.) | 5.30 (1.26; 22.25) | **0.0233** | | |
| 12 | *TMPRSS2-ERG* (yes *vs.* no) | 0.48 (0.23; 1.02) | 0.0582 | **0.0002** | n.a. |
| | *ADAMTS12* (M *vs.* U/ cont.) | >1000 | 0.9479 | | |
| 13 | *TMPRSS2-ERG* (yes *vs.* no) | 1.48 (0.73; 3.00) | 0.2748 | **0.0003** | n.a. |
| | *NAALAD2* (M *vs.* U/ cont.) | 8.57 (2.04; 35.92) | **0.0035** | | |

Selected combinations are shown. For simplicity, only models' P-values are provided for TCGA data. M/U – methylated/ unmethylated promoter status, *TMPRSS2-ERG* – fusion transcript status, pT – pathological tumor stage, pISUP – pathological ISUP grading, ISUP – International Society of Urological Pathology, PSA – prostate-specific antigen, HR – hazard ratio, CI – confidence intervals. Significant P-values are in bold, n.a. – not analyzed. *Forward entering of covariates.
